# Cost of immunization coverage surveys in low- and middle-income countries

**DOI:** 10.3389/fpubh.2026.1897118

**Published:** 2026-08-04

**Authors:** Logan Brenzel, Helen Saxenian, Katelyn Stahley, Emily Dansereau

**Affiliations:** 1 Independent Consultant, Paris, France; 2 Independent Consultant, Bethesda, MD, United States; 3 Gates Foundation, Seattle, WA, United States

**Keywords:** cost, coverage, immunization, low and middle income counties, surveys

## Abstract

**Introduction:**

Immunization coverage surveys are one of the best ways to assess program performance. However, surveys can be costly endeavors, and their costs are not well-documented, particularly in low- and middle-income countries (LMICs).

**Methods:**

This research brought together a scoping review of the literature on survey methods and costs in LMICs; qualitative interviews from a range of funders of health and immunization coverage survey; and a quantitative analysis of costs from 33 surveys. We collected and systematically categorized survey design and cost information for a range of immunization coverage surveys conducted in low- and middle-income countries between 2013 and 2025. Total and unit costs were compared across regions, survey type, survey method, and conflict setting.

**Results:**

The median total survey cost was $300,000, ranging from $28,000 to $2.87 m depending upon survey method and geographic scope. Lot Quality Assurance Sampling (LQAS) surveys had lower median total costs. The cost per household was highest in Latin American countries and lowest for East African countries. Post-campaign coverage surveys (PCCS) had the highest cost per person surveyed. Major cost drivers included personnel, transportation, and field work costs. Technical support can account for an average of 30% of survey costs.

**Discussion:**

Adequate planning and budgeting of immunization coverage surveys are essential for ensuring sufficient financing and quality implementation. Findings from this study can provide benchmarks for better budgeting and financing of immunization coverage surveys, leading to improved quality. Survey cost and budget information should be regularly analyzed and reported to enhance our ongoing knowledge.

## 1 Introduction

Immunization coverage surveys play an important role in assessing program performance and monitoring immunization coverage, particularly when reported administrative estimates may be inaccurate (1). Coverage surveys are useful in monitoring progress toward specific goals, such as reaching zero-dose children.

Adequate budgeting and planning of immunization coverage surveys are essential for ensuring sufficient financing, quality implementation and robust results, particularly in low- and middle- income countries (LMICs). The World Health Organization (WHO) has provided guidance to countries on methods for conducting immunization coverage surveys, as well as guidance on planning and budgeting for surveys (2). A detailed budget template and guidance is also part of the survey planning tools for the UNICEF Multiple Indicator Coverage Surveys (MICS) (3). The Demographic and Health Surveys (DHS) also provide budget examples and guidance (4). While these tools help with the development of survey budgets, there is limited reporting on the actual costs of preparing, conducting, analyzing and disseminating immunization coverage surveys in LMICs. One recommendation from a 2017 WHO meeting was the need to document survey budget information more consistently to understand cost drivers as well as to evaluate relationships between cost and survey designs (1).

The total costs of health and immunization surveys can be expensive depending upon the scope, complexity, and other contextual factors. One study found that DHS surveys cost $845,164 on average in Tanzania or $19.54 per participant (5). The high cost of surveys and the complexities of surveys in fragile and conflict settings have contributed to the need to find more rapid and efficient survey methods which increases the importance of understanding survey costs.

Research comparing cluster sampling methods with alternative survey designs has shown mixed results in terms of total and unit costs. One study in Nepal found that Lot Quality Sampling Approach (LQAS) was 4.5 times less expensive per household, and 5.2 times less expensive per interview than the 30 × 30 cluster sampling method (6). By contrast, an assessment in India showed that the WHO cluster method was half as expensive per cluster as the LQAS method one-third as much per household (7). In Indonesia, the cluster survey method was less costly than a random sampling approach (8); while in Ethiopia, costs were nearly equivalent, though the value of time was not factored into that analysis (9). Both studies found the WHO cluster survey method resulted in more precise estimates. Another analysis compared the time and budget for different surveys in Nepal, Bangladesh, and Vietnam. Results varied by context and method, with the gridded population survey in Nepal and the two-staged sampling survey in Vietnam having higher costs per household. Larger clusters required longer time which contributed to higher costs (10).

Survey precision, time and cost for a 30 × 30 cluster survey was compared with two alternative sampling designs in West Darfur, Sudan. While the traditional cluster survey had the highest total costs and cost per cluster, it was the least costly per household and had the highest level of precision. The alternative sampling methods required significantly less time (one-third to one-quarter) to implement (11). A comparison study of precision and costs was conducted for four survey methods (WHO multi-cluster, modified multi-cluster, a GIS-enabled method and LQAS) in the Demographic Republic of the Congo (DRC) and Central African Republic (CAR). Results show that while the WHO method had higher costs overall, it had the lowest cost per cluster across the methods. In addition, the WHO method resulted in more precise coverage estimates (12).

Previous studies on the costs of surveys have shown that personnel and field work costs account for the highest shares of total costs (10, 12, 13). Coordination costs (technical support) also may represent a large share of total costs depending upon method (12).

Results from the literature found differences in costs for different immunization coverage survey methodologies in LMICs. However, these studies utilized different methods to estimate cost hampering comparisons and limiting the utility for planning and budgeting. The objective of this research is to understand the current cost of alternative methods for immunization coverage surveys in LMICs. Research questions focus on understanding: (1) which survey methods are the most costly; (2) how survey costs vary by region and context; (3) what are the major cost drivers of immunization survey costs; (4) whether surveys conducted in fragile settings have higher or lower costs; and, (5) how indicators such as the cost per cluster, per household, and per respondent vary across survey methods. The results of this work can provide a reference point for survey budget planning and investment; improve communication between funders and implementers in negotiating survey budgets; highlight ways to reduce costs of coverage surveys; and improve overall survey quality by ensuring sufficient resources.

## 2 Materials and methods

This study used a mixed-methods approach for evaluating immunization survey costs in LMICs, including: (1) a scoping review of published and grey literature on survey costs; (2) consultations with experts from funding and implementing agencies that support immunization coverage surveys; and, (3) a quantitative analysis of coverage survey information collected from international organizations, such as the Gates Foundation, Gavi, the Inter-American Development Bank, UNICEF, the World Bank, and others.

### 2.1 Literature review

A scoping review of the published and grey literature review was conducted using three approaches: (1) online searches through PubMed; (2) web-based search on key terms; and (3) additional materials search using a snow-ball technique from references obtained through the first two strategies, covering the period 1995 to 2024.

PubMed returned 852 articles based on search terms such as coverage, surveys, questionnaires, survey methods, costs, budgets, economics, efficiency, vaccination, immunization, low- and middle-income, low income. Abstracts of these articles were reviewed by two team members and narrowed further to 10 articles that discussed survey costs, budgets, or efficiency. A full-text review proceeded to reduce the number of articles (8) which evaluated immunization coverage survey methods costs. Another PubMed search focusing on LQAS surveys resulted in 47 articles. Abstract review yielded two (2) additional articles that discussed survey costs, for a total of 10 published articles on survey costs gleaned from the search criteria.

For the second strategy, searches were conducted on Google and through many organizational websites (e.g., World Bank, WHO, UNICEF, Gavi the Vaccine Alliance, Demographic and Health Surveys (DHS), Global Financing Facility (GFF), Global Fund, 3ie, among others). Search terms used in the web-based search included: immunization coverage survey, costs, budgets, efficiency, expenditures, survey methods, phone surveys, LQAS, Post-campaign coverage surveys, LMICs, and low income.

### 2.2 Consultation of experts

A group of external stakeholders were brought together to learn of the aims of the project, provide inputs and guidance, and to review project outputs. Stakeholders included health and immunization survey funders, agencies responsible for conducting surveys, technical experts and international agency representatives. Supplementary Table S1 provides a list of the stakeholders engaged. Two team members consulted 45 experts from organizations that have a role in funding, supporting, or conducting coverage surveys. The purpose of these discussions was to identify additional surveys and budget/cost information that could be used in the analysis. Options regarding how to improve the efficiency and quality of surveys were also discussed, as well as their role in funding, supporting, developing, or conducting coverage surveys.

### 2.3 Health and immunization survey budget analysis

Immunization coverage survey design and budget information were difficult to identify in the public domain. Between February and July 2024, we obtained budget, cost, and survey design information from different organizations. Survey information was categorized and evaluated across a range of survey characteristics, survey and sample design, survey implementation details, costs and/or budgets, and financing (Table 1).

**Table 1:** Characterization factors on immunization coverage survey design and cost data.

Parameter	Details
Background information on the country and survey	When conducted, national/subnational, type of survey (immunization, post-campaign, multi-purpose), specific target population, HPV or RI, continuous or periodic, level of survey capacity, and other characteristics
Sampling frame	Number of clusters, number of households surveyed, coverage verification method (facility trace-back, card verification), enumeration required
Survey implementation	Number of teams, persons per team, number of households, time per interview, level of TA and coordination, financing
Cost elements	Salaries, per diem, transport, supplies, equipment, coordination, printing, contingency/other, capital costs, technical assistance and coordination
Quality of the survey	Sampling frame maintained, target population specified, pre-testing, measures to limit response and non-response bias, confidentiality measures undertaken, where possible. Survey quality could not be consistently appraised given information constraints.
Survey financing	Funding sources, funding amounts, timing of funds released
Other	Contact person, email, institution, other qualitative information

The initial sample covered 59 surveys from 27 countries. Sixty-three percent of surveys were from the Africa Region, evenly split between West and East Africa (based on UNICEF country classifications). There were nine surveys from one East African country, and six surveys from a West African country.

The quantitative analysis of immunization coverage survey costs was conducted using Excel v16.110. Surveys analyzed included the WHO Vaccination Coverage Cluster Survey methodology (14); Lot Quality Assurance Sampling (LQAS) (15–17); and Post Campaign Coverage Surveys (PCCS) used to measure coverage following a supplementary immunization campaign (18). The exercise also examined broader health surveys such as the Multiple Indicator Cluster Surveys (MICS) and phone surveys.

The survey sample was highly variable in nature. To make comparisons, we limited our analysis to a subset of 33 surveys. The subsample excluded small pilot studies and serosurveys. We also excluded those surveys that were conducted in limited geographic areas. The final sample of 33 surveys evaluated national immunization coverage. This framing represented a more homogeneous sample from which to conduct the descriptive analysis. For the analysis, we generated averages, medians, and inter-quartile ranges for a set of indicators: total costs, cost per cluster, cost per household and cost per person surveyed. These indicators were evaluated by region, survey methodology, and conflict setting. Assessment was also made to determine the major cost drivers or the proportional shares of the total cost for different line items. Regression analysis was not done because of the small sample size.

Local currency estimates were converted into US dollars using the average exchange rates for the relevant year. For comparison purposes, costs reported in different years were converted to constant 2023 US$ based on the world Consumer Price Index from the World Bank’s World Development Indicators database (19). Categorization of conflict countries was sourced from the United Nations (20) and population density estimates also were obtained from the World Bank (19).

## 3 Results

This section focuses on results from the quantitative analysis and stakeholder interviews.

### 3.1 Quantitative analysis

The analysis sample of 33 surveys had the following characteristics. Nearly 80% of the surveys were from African countries: 60% of the surveys were from countries in the West and Central African Region (WCARO), followed by 18% in the East and Southern African Region (ESARO), and 12%from the Eastern Mediterranean Region. The Democratic Republic of the Congo (DRC) and Nigeria each represented 15% of the sample. More than half of the subsample was from a fragile or conflict-affected country. In terms of survey methods, 21% of surveys utilized multi-stage sampling methods recommended by the WHO. Post-campaign coverage and MICs surveys account for 24% of the subsample. Phone and LQAS surveys accounted for 15% each of the subsample. Routine immunization coverage surveys represented one-third, and polio coverage specific surveys accounted for 9% of the subsample.

Survey costs had a right-tailed distribution and median values are compared. Table 2 shows that the subsample had a median total cost of approximately $300,000 (IQR: $154,670 - $1.48 m). The median cost per cluster was $1,135 (IQR: $888 - $1,723), and the median cost per household was $86 (IQR: $22 - $149). The median cost per respondent was $145 (IQR: $105 - $174). The median cost per household for the subsample was statistically significantly lower than the median cost ($96) in the initial sample of 59 surveys (*p* < 0.05). Total cost, cost per cluster and cost per respondent were not significantly different between the initial sample and the subsample.

**Table 2:** Summary statistics for the sample of surveys generating national-level estimates (US$ 2023).

Indicator	Total cost	Cost/household	Cost/cluster	Cost/respondent
Average	$816,958	$104	$1,339	$141
Median	$298,567	$86	$1,135	$145
q1	$154,670	$22	$888	$105
q3	$1,482,387	$149	$1,723	$174
Count	33	19	18	18

**Figure 1:**
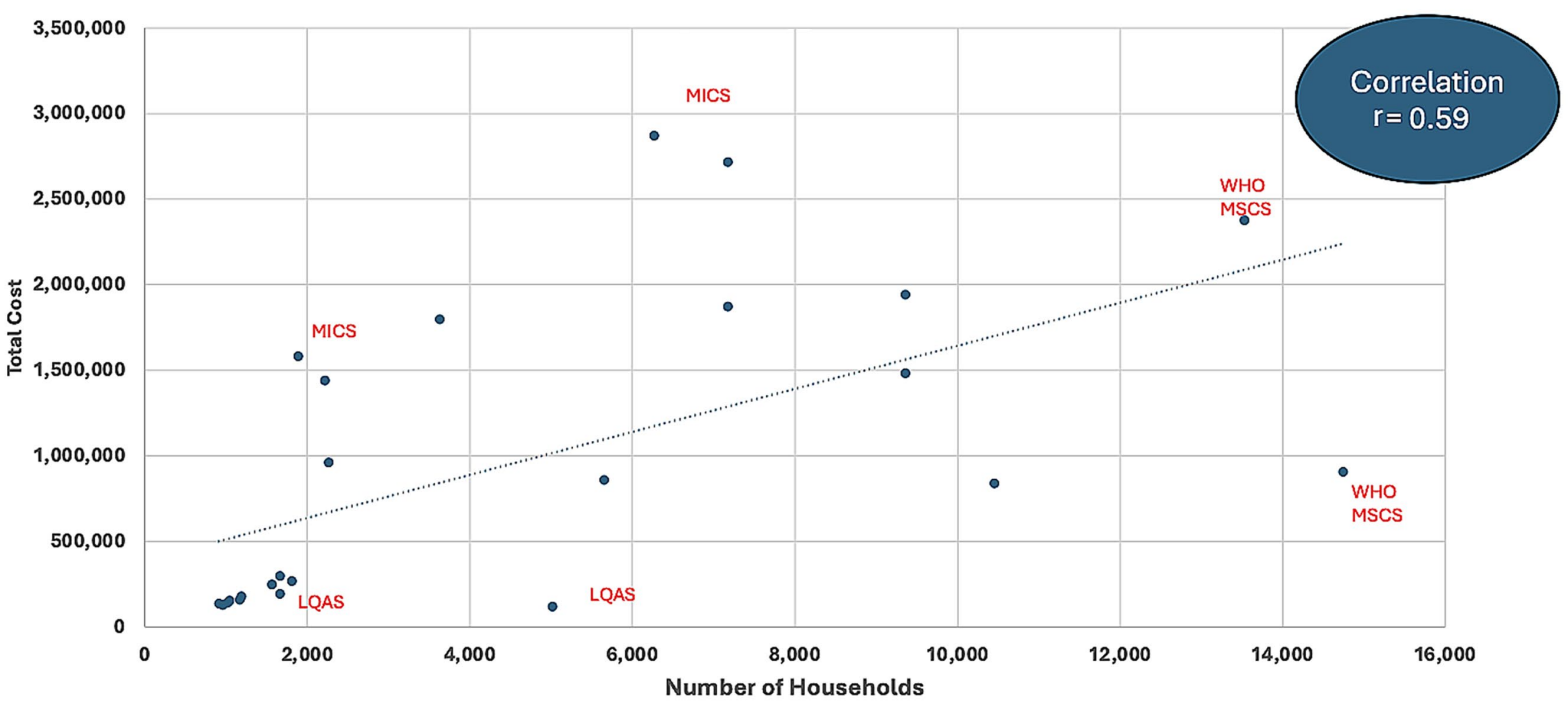
Relationship between survey size and total cost (national level, *n* = 19).

There was a moderate positive correlation between the number of households surveyed and total survey cost (*r* = 0.58). The cost per cluster was negatively correlated with the number of clusters (*r* = −0.2) suggesting that fixed costs of surveys are spread over larger numbers of clusters. While there was no correlation between total survey cost and population density, there was a moderate positive relationship (*r* = 0.59) between population density and the cost per respondent interviewed. The greater the number of survey households, the more costly that survey becomes (see Figure 1).

Regional variations were found in the subsample of national level estimates. The median total cost was highest for surveys conducted in the PAHO region ($1.8 m; IQR: $1.79 m - $2.33 m) followed by those from the EMRO region ($1.4 m; IQR: $754,661 - $2.08 m). Surveys from the ESARO subregion of Africa had the lowest median total costs ($210,000; IQR: $137,764 - $254,481). These patterns were similar for the cost per household, with PAHO surveys having the highest value ($155; IQR: $147 - $164) which was more than ten times that for ESARO region surveys ($14: IQR: $12 - $14). Surveys in the PAHO region were largely multi-health topic and the ESARO surveys included many lower-cost phone surveys which could explain the differences. Surveys in the EMRO region had the highest median value for the cost per respondent ($150; IQR: $128 - $153) followed by surveys in WCARO ($142; IQR: $100 - $165). The median cost per cluster was highest for WCARO surveys ($1,307; IQR: $761 - $1,796) and the lowest median cost/cluster was found for the ESARO surveys ($984; IQR: $1,035 - $1,138). The cost/household was significantly different between ESARO and WCARO surveys (*t* = 0.004) as was the number of households surveyed (*t* = 0.003) (see Table 3).

**Table 3:** Regional comparisons for national level immunization coverage surveys (US$ 2023).

Region	Indicator	Total cost	Cost/household	Cost/cluster	Cost/respondent
EMRO	Average	$ 1,421,739	$ 183	$1,011	$157
	Median	$ 1,416,303	$ 107	$1,051	$150
	Q1	$ 754,661	$ 85	$555	$128
	Q3	$ 2,083,381	$ 243	$1,486	$183
ESARO	Average	$ 523,592	$ 17	$1,010	$98
	Median	$ 210,802	$ 14	$984	$97
	Q1	$ 137,764	$ 12	$1,035	$97
	Q3	$ 254,481	$ 14	$1,138	$155
PAHO	Average	$ 2,150,410	$ 155	n/a	n/a
	Median	$ 1,797,854	$ 155	n/a	n/a
	Q1	$ 1,790,025	$ 147	n/a	n/a
	Q3	$2,334,518	$ 164	n/a	n/a
WCARO	Average	$702,868	$ 98	$1,460	$138
	Median	$298,567	$ 86	$1,307	$142
	q1	$151,901	$ 30	$761	$100
	q3	$1,172,493	$ 137	$1,796	$165
WPRO		$178,350		$1,372	$196

Information concerning the number of clusters and respondents for the surveys in the PAHO region were unavailable to us.

Table 4 illustrates the results by survey method for the subsample. The lowest median total cost is for PCCS ($147,548) and LQAS surveys ($144,321), while MICS and WHO methods are the more costly surveys ($1.79 m and $905,201, respectively). MICS surveys often assess other priority health interventions so there is potential added value for these combined surveys that are not evaluated here. Differences in median total cost between WHO multi-staged cluster sampling and LQAS are statistically significant (*t* = 0.03), although we cannot draw definitive conclusions because of the small sample size. While the LQAS and WHO multi-stage cluster surveys have lower median cost per household ($84 and $86), they have higher values for the median cost per cluster ($1,584 and $1,122, respectively). MICS surveys had the highest cost/person surveyed ($204; IQR: $170 - $232), followed by PCCS ($152; IQR: $142 - $171).

**Table 4:** Cost of survey methods that generate national level immunization coverage estimates (US$ 2023).

Method	Indicator	Total cost	Cost/household	Cost/cluster	Cost/respondent
LQAS (*n*=5)	Average	$146,845	$86^a^	$1,122^a^	$86^a^
	Median	$144,321	$86	$1,122	$86
	q1	$118,006	$86	$799	$86
	q3	$194,682	$86	$1,446	$86
MICS (*n*=8)	Average	$1,877,906	$190	$991	$198
	Median	$1,790,025	$172	$991	$204
	q1	$1,546,337	$123	$525	$170
	q3	$2,083,381	$236	$1,456	$232
PCCS (*n*=8)	Average	$162,014	n/a	$988	$148
	Median	$147,548	n/a	$1,051	$152
	q1	$134,843	n/a	$991	$142
	q3	$164,198	n/a	$1,135	$171
Phone (*n*=5)	Average	$240,511	$13	n/a	n/a
	Median	$210,802	$14	n/a	n/a
	q1	$160,160	$10	n/a	n/a
	q3	$305,441	$15	n/a	n/a
Who multi-stage sampling (*n*=7)	Average	$1,243,353	$84	$1,851	$97
	Median	$905,201	$84	$1,584	$105
	q1	$848,855	$38	$1,196	$95
	q3	$1,712,721	$113	$2,614	$109

a Sample size for these indicators reduced to one observation, resulting in no difference between indicators.

Survey budgets in the subsample contain different numbers and nomenclature for line items making comparisons of cost drivers challenging. We report median values which are not additive for the most frequently utilized line items. For the surveys that included economic classification line items such as personnel, per diem, or transportation, they accounted for 31, 20 and 10% of total costs, respectively. Among those surveys that categorized costs according to activities such as field work, logistics, training, and analysis and reporting costs, these accounted for 58, 25, 13 and 16%, respectively. The median share of technical support costs was relatively high at 32% of total costs in the subsample.

### 3.2 Limitations

There were several caveats and limitations to the quantitative analysis. The findings of this study reflected the information that could be collected within the time-period available for the project. The sample of surveys contained limited information for WPRO and SEARO regions, and the sample contained information primarily from the Africa region which affects broader generalizability. We were unable to obtain information for Demographic and Health Surveys (DHS) because of their confidential nature. The number of surveys that could be analyzed by indicator varied which may have resulted in a higher median cost per person surveyed than the median cost per household. Survey budgets also did not include government contributions, and this remains a gap in our knowledge. We also have no consistent knowledge of whether budgets were fully used, under-estimated, or overestimated. Finally, many of the survey budget estimates were provided to us in confidence and the analysis was accomplished to maintain anonymity which may limit the detail that can be provided.

## 4 Discussion

This study represents one of the first attempts to collect and systematically examine the costs of immunization coverage surveys across various contexts and methodologies. Improving the quality and availability of survey cost information should be a priority to help inform the true resource needs for conducting quality immunization coverage surveys.

Improving survey efficiency is a priority for future immunization coverage surveys, given the dramatic recent declines of 23% in official development assistance (ODA) to health which can affect funding technical support and conduct of surveys (21). Use of satellite data to estimate population densities and set enumeration areas may be a potentially cost-saving innovation (22–24). Pooling resources and combining surveys can lead to greater efficiency. One example is the Nigeria MICS/NICS which nested a national immunization coverage survey into a MICS survey (25). In addition, conducting surveys too close in time with each other may not be the best use of scarce resources. Optimal phasing and timing of surveys can be cost saving (26). Adding a new module to an established survey can impact survey customization, length, sample size, training, supervision, and data processing which may add costs of implementing the survey. UNICEF is working toward understanding the incremental costs of adding modules into its MICS surveys (27).

Shortening field work time has been shown to be cost saving (7, 8, 11, 22). Embedding local data collectors in field teams may save on costs. The complexity of vaccination verification techniques may drive up costs and efforts to reduce the time involved may create efficiencies. Taking photos of immunization cards in the field and uploading them for centralized transcription could save interviewer and respondent time and potentially improve cost and quality (27).

Phone surveys may be a less costly way of quickly determining whether a child has received a particular vaccine but there may be a tradeoff with quality and precision. Phone surveys have been used to measure COVID19 vaccination (28). However, for childhood vaccination, this method may not provide the most accurate information if respondents are not caregivers and cannot locate and/or decipher immunization cards.

Technical support costs were found to be an important cost driver in our analysis. In many cases, technical support is essential if a country has not conducted a coverage survey previously or for a long period of time, or if new sampling and survey tools are being introduced such as gridded sampling (29). Using local expertise and undertaking cross-country capacity building around the use of new technological tools can be cost saving as well.

Many international partners and governments make large investments in immunization coverage surveys either as stand-alone immunization surveys or as part of broader health surveys. Nearly 40% of the sample surveys were fully or partially funded by the Gates Foundation. These included surveys as part of head-to-head survey methodology comparisons, including LQAS and large national surveys representative at the district level. Experts interviewed noted that coverage survey financing can be highly fragmented leading to delays in survey implementation and higher transactions costs for countries, impacting timeliness, quality and cost of surveys. Respondents also indicated that data processing and analysis, airtime for communication during field work, community engagement, and security in conflict settings can often be under-budgeted. The level of government support for surveys is often unknown. Having better financing information up front should lead to better survey execution.

Better and more consistent data on the cost of surveys can help inform decision-makers about the likely costs of different survey approaches. There is a need for greater standardization of budget categories and formats to support comparability. Ensuring greater transparency and reporting of survey budgets in final survey reports as a standard annex would contribute to our knowledge on immunization survey costs. Understanding the full costs of immunization coverage surveys based on actual expenditures and the value of time may provide a better window into resource needs for planning and budgeting quality surveys.

## 5 Conclusion

Total global immunization spending topped $10 billion by 2017 (30). Given the large investment, immunization coverage surveys can play a pivotal role in strengthening program performance and improving the efficient use of resources.

This study represents one of the first attempts to describe the cost of immunization coverage surveys across geographies and different methodologies. This analysis raises other questions to be addressed by further research. Understanding the marginal costs of adding clusters or districts to a survey will be important for planners and funders to determine the survey scope within an overall budget constraint. Evaluating costs against survey precision can guide decisions around survey and sampling design. Understanding the cost and cost savings of specific coverage survey innovations could also help guide decisions on investments (31). Future analysis could focus on analyzing funding sources and timeliness of disbursements for coverage surveys, with a particular focus on government contributions. Finally, it will be important to analyze surveys efficiencies and potential areas for cost savings.

## Data Availability

The original contributions presented in the study are included in the article/Supplementary material, further inquiries can be directed to the corresponding author.
