# Supplementary material for: Cost of immunization coverage surveys in low- and middle-income countries
Source: Front Public Health. 2026 Aug 4;14:1897118. doi: 10.3389/fpubh.2026.1897118 (PMC13481263; doi:10.3389/fpubh.2026.1897118)
Supplement: Supplementary file 1 [file Table_1.DOCX]

**Table S1: Stakeholder organizations participating in qualitative interviews**

| Organization | Role in Immunization Coverage Surveys |
| --- | --- |
| Gavi | Funder of post-campaign coverage surveys and other immunization coverage methods. |
| Gates Foundation | Funder of large national surveys evaluating coverage and zero dose children. |
| World Health Organization | Technical support, guidance and funding for immunization coverage surveys. |
| UNICEF | Technical support and funding for the Multiple Indicator Coverage Surveys (MICS). |
| USAID | Former funder of the Demographic and Health Surveys. |
| World Bank | Funder and technical support and guidance for the Living Standard Household Study (LSMS) household survey costs and phone surveys. |
| Inter-American Development Bank | Conduct of coverage evaluations of the Salud MesoAmerica Initiative (SMI). |
| Global Financing Facility | Technical support and funding of health facility phone surveys. |
| US Centers for Disease Control | Technical support and funding for immunization coverage surveys. |
| Other funding and implementing organizations and independent consultants | BioStat Consulting, 3ie, Global Fund, Aga Khan University, the Johns Hopkins Bloomberg School of Hygiene and Tropical Medicine, Johns Snow, Inc., Liverpool School of Tropical Medicine, University of Washington, ICF International. |
